# Proliferative Pathways of Vascular Smooth Muscle Cells in Response to Intermittent Hypoxia

**DOI:** 10.3390/ijms20112706

**Published:** 2019-06-01

**Authors:** Yoji Kyotani, Shin Takasawa, Masanori Yoshizumi

**Affiliations:** 1 Department of Pharmacology, Nara Medical University School of Medicine, Kashihara 634-8521, Japan; yoshizu@naramed-u.ac.jp; 2 Department of Biochemistry, Nara Medical University School of Medicine, Kashihara 634-8521, Japan; shintksw@naramed-u.ac.jp

**Keywords:** intermittent hypoxia, vascular smooth muscle cells, epiregulin, interleukin

## Abstract

Obstructive sleep apnea (OSA) is characterized by intermittent hypoxia (IH) and is a risk factor for cardiovascular diseases (e.g., atherosclerosis) and chronic inflammatory diseases (CID). The excessive proliferation of vascular smooth muscle cells (VSMCs) plays a pivotal role in the progression of atherosclerosis. Hypoxia-inducible factor-1 and nuclear factor-κB are thought to be the main factors involved in responses to IH and in regulating adaptations or inflammation pathways, however, further evidence is needed to demonstrate the underlying mechanisms of this process in VSMCs. Furthermore, few studies of IH have examined smooth muscle cell responses. Our previous studies demonstrated that increased interleukin (IL)-6, epidermal growth factor family ligands, and erbB2 receptor, some of which amplify inflammation and, consequently, induce CID, were induced by IH and were involved in the proliferation of VSMCs. Since IH increased IL-6 and epiregulin expression in VSMCs, the same phenomenon may also occur in other smooth muscle cells, and, consequently, may be related to the incidence or progression of several diseases. In the present review, we describe how IH can induce the excessive proliferation of VSMCs and we develop the suggestion that other CID may be related to the effects of IH on other smooth muscle cells.

## 1. Introduction

**Figure 1:**
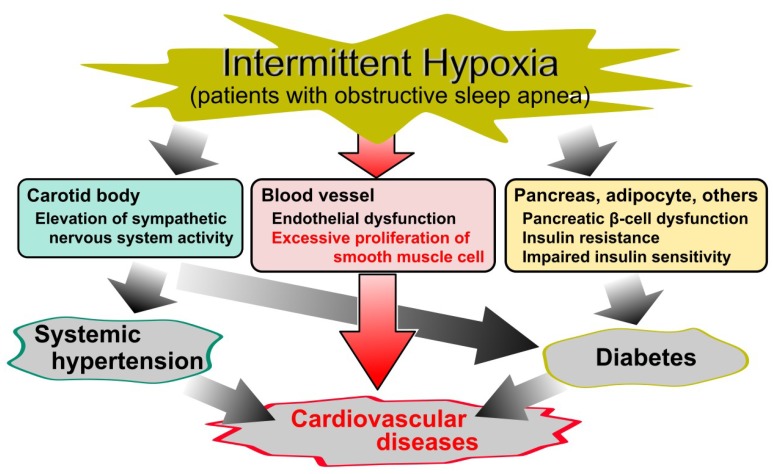
Cause and effect diagram of obstructive sleep apnea (OSA)-related diseases. Although intermittent hypoxia (IH) in OSA is a known risk factor for diabetes, systematic hypertension, and cardiovascular diseases, the cellular mechanisms underlying the relationship between IH and cardiovascular diseases remain elusive. Despite a large number of studies of IH, the molecular mechanism of IH on vascular smooth muscle cells is less established.

Obstructive sleep apnea (OSA) is characterized by repeated episodes of intermittent hypoxia (IH), i.e., transient oxygen (O_2_) desaturation, and resaturation. In clinical practice, OSA is commonly diagnosed by polysomnography and its severity is classified by the apnea hypopnea index (AHI) as follows: mild, AHI ≥5; moderate, AHI ≥15; severe, AHI ≥30 [1,2]. It is a highly prevalent disorder [3,4]; Peppard et al. estimated that the prevalence of moderate to severe sleep-disordered breathing is 10% and 3% among 30- to 49-year-old men and women, respectively, and 17% and 9% among 50- to 70-year-old men and women, respectively [3]. Furthermore, OSA is well known as a risk factor for diabetes, systematic hypertension, and cardiovascular diseases [5,6,7,8,9,10,11,12,13,14,15,16], and also increases mortality from cardiovascular diseases (Figure 1) [17,18].

Continuous positive airway pressure (CPAP) is a clinically effective strategy for treating several diseases that derive from OSA. A number of studies have shown that CPAP decreases hemoglobin A1c levels, blood pressure, and inflammatory markers, as well as the frequency of cardiovascular events [19,20,21,22]. However, some studies have reported no significant effects of CPAP on glycemic control, serum lipids, hypertension, or cardiovascular events [23,24,25,26]. Additionally, patient compliance with CPAP treatment is often unsatisfactory [27,28,29]. Therefore, a clarification of the mechanisms underlying atherosclerosis in response to IH is important for establishing prophylaxis against OSA-related diseases.

Atherosclerosis is well known as a major risk factor for cardiovascular diseases that can result in heart diseases and stroke. It is characterized by the formation of lesions, foam cells, and fibrous plaques. The major features in the progression of atherosclerosis are inflammation, the dysfunction of the endothelial barrier, oxidative stress, and the excessive proliferation of vascular smooth muscle cells (VSMCs) [30,31]. However, the pathophysiology of these cardiovascular diseases in OSA remains incompletely understood. OSA-related cardiovascular diseases are generally thought to be caused by various pathophysiological triggers, such as sympathetic nervous system overactivity, systemic inflammation, and oxidative stress, which in turn lead to metabolic dysregulation, hypertension, and endothelial dysfunction [32,33]. In vitro and in vivo models of IH have allowed researchers to investigate the influences of IH on several tissues and cells, and although articles on the vascular effects in IH and cardiovascular diseases in OSA syndrome have been previously published, the effects of IH on VSMCs, including its molecular mechanisms, have not been described [14,15]. Furthermore, there are few in vitro or in vivo studies of IH in other smooth muscle cells.

Recently, our laboratory demonstrated that IH directly increased the number of VSMCs by increasing the epidermal growth factor (EGF) family ligands and the EGF receptor erbB2, which were partially mediated by the IH-induced increase of interleukin (IL)-6 [34,35]. In the present review, we summarize the effects of IH on VSMCs, focusing on the intracellular mechanisms related to atherosclerosis, and develop a discussion of other chronic inflammatory diseases (CID).

## 2. Vascular Smooth Muscle Cells (VSMCs) in Atherosclerosis

Typically, VSMCs have been regarded as key players in the progression of atherosclerosis because their excessive proliferation promotes plaque formation, and then their presence in the advanced plaques prevent the rupture of the plaques’ fibrous caps. VSMCs in normal arterial media have a spindle shape, termed the contractile phenotype, however, in damaged vessels, VSMCs develop a proinflammatory phenotype that produces proinflammatory mediators responsible for proliferation and chemotaxis. Thus, in both beneficial and detrimental ways, inflammatory responses and an excessive proliferation of VSMCs are responsible for the progression of atherosclerosis [36,37].

## 3. Reactive Oxygen Species (ROS) and Transcriptional Factors in Intermittent Hypoxia (IH)

A large number of previous in vivo and in vitro studies have shown that IH-induced intracellular mechanisms are mainly classified into two different transcription pathways, where the hypoxia-inducible factor (HIF)-1 and the nuclear factor (NF)-κB play central roles [16,33,38]. In carotid bodies, IH-induced ROS generation is associated with HIF-1 activity and results in a sensory long-term facilitation of carotid bodies [39,40,41]. Several in vivo and in vitro studies have also observed that IH induces the activation of NF-κB in cardiovascular tissues and endothelial cells [42,43,44,45]. Our previous research, using reporter gene assays, also confirmed that IH induces the activation of NF-κB in cultured rat aortic smooth muscle cells (RASMCs) [34]. Taken together, all of these studies suggest that IH activates alternative transcriptional pathways depending on the tissue and cell types. Similarly, Kaczmarek et al. showed that IH decreased HIF-1α expression in human dermal microvascular endothelial cells but increased HIF-1α expression in human coronary artery endothelial cells, indicating that endothelial cells in cultures originating from distinct vascular beds respond differently to IH stress [46].

### 3.1. Reactive Oxygen Species (ROS)

ROS, such as superoxide anion, hydrogen peroxide, and hydroxyl radical, are well known as products of a partial reduction of oxygen. They are generated either in the processes of mitochondrial oxidative phosphorylation or during cellular responses to exogenous sources. Excessive ROS cause oxidative stress, which in turn results in macromolecular damage and is implicated in various diseases, including atherosclerosis [47]. In fact, it has been suggested that ROS are generated in patients with OSA [38,48,49,50,51] and are associated with the pathogenesis of cardiovascular diseases [52]. Furthermore, it has also been found that the mRNA molecules of heme oxygenase 1, superoxide dismutase (SOD) 1 and 2, and catalase, which are all involved in the modulation of ROS, are also changed in patients with OSA [53].

Makarenko et al. conducted an in vitro study using human lung microvascular endothelial cells and found that IH increased ROS levels and led to the reorganization of cytoskeleton and junction proteins via the ROS-dependent activation of p38 mitogen-activated protein kinases (MAPK), which resulted in endothelial barrier dysfunction [54]. Similarly, Recoquillon et al. used human aortic endothelial cells and also reported that IH increased ROS and nitric oxide production, p65-NF-κB activation, and IL-6 secretion [44]. In contrast, Hoffmann et al. found that, in human coronary artery endothelial cells, IH increased manganese SOD activity via an increased dual-specificity phosphatase 1 (DUSP1) expression, and that overnight IH induced the expression of DUSP1 in mononuclear cells and granulocytes from patients with OSA [55]. Therefore, there is some evidence that ROS play a pivotal role in mediating the cardiovascular pathology associated with IH.

However, Hayakawa et al. suggested that ROS were unlikely to mediate the activation of NF-κB [56], while Ryan et al. did not detect any influence on NF-κB activation from the presence of ROS scavenger N-acetyl-L-cysteine [43]. With respect to VSMCs, few studies have examined the involvement of ROS in IH. In our previous study, TEMPOL (1-oxyl-2,2,6,6-tetramethyl-4-hydroxypiperidine), a SOD mimic, did not exhibit any inhibitory effects on the IH-induced proliferation of RASMCs [34]. Therefore, further investigation is needed to elucidate the roles of ROS in VSMC responses in IH.

### 3.2. Nuclear Factor (NF)-κB

The eukaryotic transcription factor, NF-κB, is a key mediator involved in the control of a large number of cellular processes, especially in immune and inflammatory responses [57,58]. In the NF-κB activation pathway, degradation of the inhibitor of NF-κB (I-κB) results in the translocation of NF-κB to the nucleus. This in turn causes an increase of inflammatory cytokines, such as IL-6 and IL-8 [59,60].

A number of in vivo and in vitro studies have found that IH activates NF-κB in cardiovascular tissues and endothelial cells [42,44,45]. Ryan et al. demonstrated that IH activates NF-κB, rather than HIF-1α, via p38 MAPK, in both HeLa cells and bovine aortic endothelial cells [43,61]. The well-known stress-activated protein kinase, p38 MAPK, is frequently activated by a wide range of environmental stresses and cytokines and induces inflammation [62]. Therefore, it seems likely that p38 MAPK is a key player in the IH-induced activation of NF-κB in cardiovascular tissues and endothelial cells. However, although the IH-induced activation of NF-κB has been observed in RASMCs [34], we did not find the phosphorylation of p38 MAPK in IH for 24 h [63]. We did, however, confirm the transient phosphorylation of extracellular signal-regulated kinase (ERK) 1/2 and protein kinase B (Akt) induced by IH in RASMCs [63]. Imano et al. also found that IH increased the expression of ERK1/2 and NF-κB in human cardiac microvascular endothelial cells [45]. Taking into account the relationships among ERK1/2, Akt, HIF-1, and NF-κB [64,65,66], these factors, in addition to p38 MAPK, may also play important roles in the IH-induced activation of NF-κB.

### 3.3. Hypoxia-Inducible Factor (HIF)-1

HIFs are well-characterized transcriptional factors that are one of primary regulators of oxygen homeostasis in every cell of the body. IH exposure creates an imbalance between the activities of HIF-1 and HIF-2 via ROS generation, which leads in turn to oxidative stress, resulting in pathological states like hypertension and breathing abnormalities [16,38]. It has been well established that IH induces the activation of HIF-1 in carotid bodies. However, few studies have shown any IH-induced activation of HIF-1 either in other tissues and cells or in vascular smooth muscles. Polotsky et al. suggested that, in human aortic endothelial cells, IH (16% and 0% O_2_) and sustained hypoxia (4% O_2_) induced the mRNA expression of antioxidant genes, including heme oxygenase-1 and nuclear factor (erythroid-derived 2)-like 2 (NRF2), excluding HIF-1-related genes, such as endothelin and glucose transporter (GLUT)1 [67]. Furthermore, Kaczmarek et al. demonstrated that IH-induced changes of HIF-1α expression were quite different between endothelial cells in cultures originating from distinct vascular beds [46]. On the other hand, our study of RASMCs found that sustained hypoxia (1% O_2_) induced a large increase of GLUT1 mRNA and IH which led to a very slight increase of GLUT1 mRNA [63]. Furthermore, IH has been shown to significantly increase IL-6 expression, which is mainly mediated by NF-κB rather than HIF-1 [35]. In conjunction with the studies showing the involvement of NF-κB in IH, these results suggest that, in VSMCs, HIF-1 has more difficulty functioning in response to IH, as compared with NF-κB . Thus, NF-κB, but not HIF-1, probably plays an important role in VSMC responses in IH.

### 3.4. Interaction Between Nuclear Factor (NF)-κB and Hypoxia-Inducible Factor (HIF)-1

**Figure 2:**
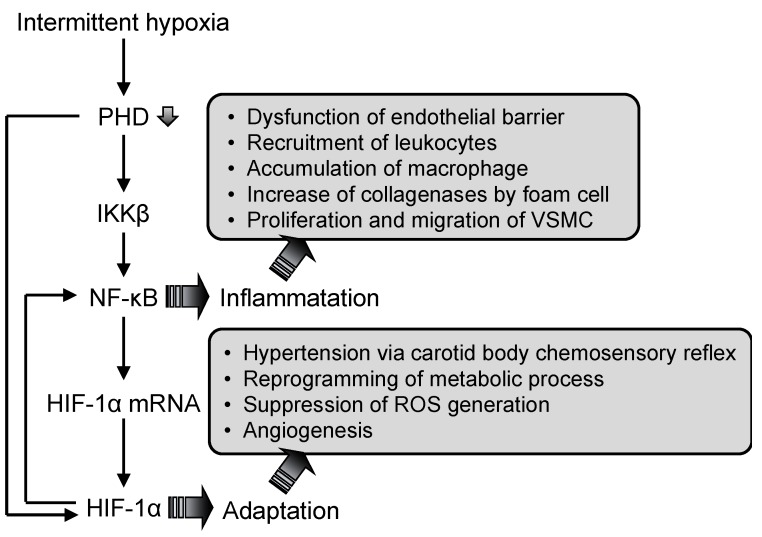
Model of the hypoxia-inducible factor (HIF)-1 and nuclear factor (NF)-κB activation mechanisms in response to intermittent hypoxia (IH). IH-induced hypoxic condition decreases PHD activity. As a result, NF-κB is induced to activate via the activation of I-κB kinase-β (IKKβ), which activates both the NF-κB mediated inflammation pathway and the NF-κB mediated up-regulation of HIF-1. Activation of NF-κB and HIF-1 induces inflammation and adaptation to IH, resulting in angiogenesis via the proliferation and migration of VSMC. PHD: prolyl hydroxylases.

A significant relationship between NF-κB and HIF-1 has been established, along with their independent roles in hypoxia. The NF-κB binding site is located in the promoter region of the HIF-1 gene, and NF-κB regulates basal HIF-1α expression [68,69]. I-κB kinase-β (IKKβ), whose catalytic activity is repressed by O_2_-sensitive prolyl hydroxylases (PHDs), is a key factor in the mRNA expression of HIF-1α [64,70]. It has also been demonstrated that HIF-1 regulates NF-κB activity in human peripheral blood neutrophils, and that the overexpression of HIF-1 results in an increased NF-κB activity and an enhanced inflammatory response in HIF-1 transgenic mice [71,72]. Therefore, NF-κB and HIF-1 are thought to have interdependent roles and pathways that are important for modulating inflammatory responses to intermittent hypoxia (Figure 2) [73,74]. Furthermore, the susceptibility between NF-κB and HIF-1, depending on cells, may cause the different response to IH.

## 4. Interleukin (IL)-6

Several clinical studies have demonstrated a significant correlation of OSA with inflammatory markers, such as C-reactive proteins, interleukins, intracellular adhesion molecules, and tumor necrosis factor-α [75,76,77,78]. These studies indicated that IH in patients with OSA induces systemic inflammation, which is involved in the progression of atherosclerosis. In fact, in a meta-analysis of 29 population-based prospective studies, IL-6 and IL-18 were associated with increases in the adjusted relative risks for nonfatal myocardial infarctions and coronary heart disease deaths [79].

Our in vitro model confirmed that IH increased IL-6 in human coronary artery smooth muscle cells (hCASMCs) [35]. Similarly, increases of IL-6 and IL-8 with IH have been observed in both human endothelial cells and cardiac myocytes [44,67,80]. These results suggest that IH causes inflammation in vessel walls or proximal tissues, since IL-6 and IL-8 function as autocrine/paracrine inflammatory cytokines. The IL-6 amplifier may also be present, due to the simultaneous activations of the nuclear factor NF-κB and the signal transducer and activator of transcription 3 (STAT3), which increases the expression of chemokines in non-immune cells and induces inflammation via a NF-κB loop, resulting in the accumulation of various immune cells and the dysregulation of homeostasis. The IL-6 amplifier is also thought to be associated with a number of diseases and disorders [81,82,83] as well as cardiovascular diseases, including atherosclerosis, in patients with OSA, since IH activates both NF-κB (via hypoxic conditions) and STAT3 (via increased IL-6) [34,35,43,61,83].

In addition to inflammation, the creation of macrophage foam cells is also an important feature of the progression of atherosclerosis. Likely, IL-6 partially contributes to increases in major scavenger receptors such as scavenger receptor A and CD36, in macrophages, and likely induces macrophage foam cell formation [84]. Given the increases of IL-6 from VSMCs and other vascular tissues, IH may facilitate macrophage foam cell formations in lesions, and thereby contribute to the progression of atherosclerosis.

## 5. Epiregulin

Epiregulin belongs to the EGF family and is expressed as type 1 transmembrane precursors, which are cleaved by disintegrin and metalloproteinase enzymes to release mature forms. The mature growth factors bind to members of the erbB family of receptor tyrosine kinases to regulate the proliferation, differentiation, and variation of mature cell functions. Epiregulin also plays an important role in angiogenesis and vascular remodeling, particularly during inflammation [85,86,87].

Takahashi et al. showed that epiregulin is released from ERK1/2- and p38 MAPK-activated VSMCs and it acts as a major autocrine/paracrine factor for VSMC dedifferentiation, and thus proliferation, suggesting that epiregulin regulates vascular remodeling such as atherosclerosis [88]. Similarly, our previous study found that IH induced the proliferation of VSMCs via an increase of epiregulin [34]. We confirmed that the phosphorylation level of ERK1/2 was significantly increased by IH and then became a decreased level as compared with that of normoxia in RASMCs, and that IH increased DUSP1 mRNA in RASMCs [63,89]. Hoffman et al. also reported an IH-induced increase of DUSP1 expression in human coronary artery endothelial cells [55]. Since DUSP1 is one of the mitogen-activated, stress-inducible and dual-specificity MAPK phosphatases [90], the imbalance between ERK1/2 and DUSP1 activities likely contributes to the IH-induced increase of epiregulin expression in VSMCs.

**Figure 3:**
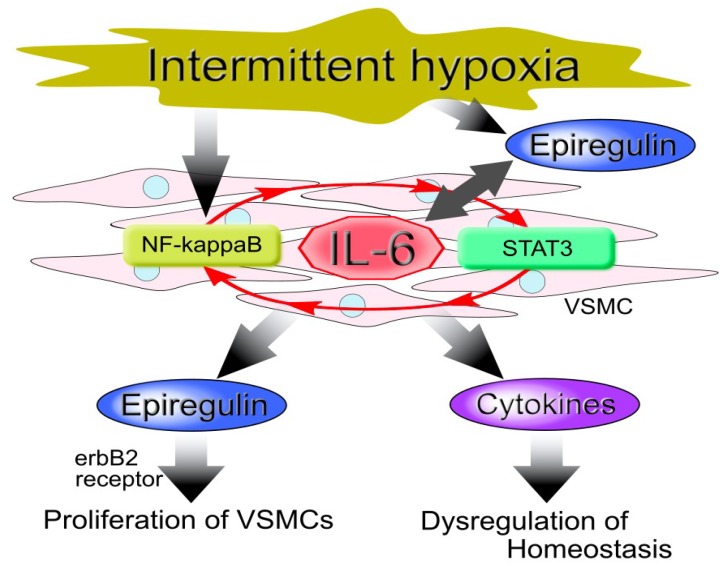
Model of vascular smooth muscle cell (VSMC) cellular responses to intermittent hypoxia (IH). IH induces the up-regulation of interleukin (IL)-6 via activation of the nuclear factor (NF)-κB and the signal transducer and activator of transcription 3 (STAT3), which involves the IL-6 amplifier (red arrow). This results in an increased expression of epiregulin and other cytokines that proliferate VSMCs, leading to atherosclerosis.

With respect to the IL-6 amplifier, Murakami et al. demonstrated the involvement of epiregulin in the development of inflammatory diseases [91]. However, we found that IH up-regulated IL-6 which in turn increased epiregulin expression in hCASMCs [35]. These results suggest that IH-induced IL-6 and epiregulin cooperatively induce inflammation, resulting in the dysregulation of homeostasis in the vessel tissues of patients with OSA (Figure 3).

Interestingly, in our promoter assay using VSMCs, where an epiregulin promoter-luciferase reporter were transiently expressed, IH exhibited no significant effect on epiregulin promoter activity despite its up-regulation of epiregulin mRNA [35]. This suggests that the IH-induced increase of epiregulin mRNA does not depend on transcriptional activation, including any activation of NF-κB and HIF-1. Therefore, the underlying mechanisms in the IH-induced increase of epiregulin remain unclear and are very attractive areas for future research to establish prophylaxis for cardiovascular diseases in patients with OSA. Recently, there has been increased evidence that IH induces changes in microRNA expression in several types of cells in patients with sleep disorders [92,93,94,95], and therefore microRNAs may play pivotal roles in cellular responses to IH, including the increase of epiregulin expression in VSMCs.

## 6. Chronic Inflammatory Diseases (CID)

IL-6 and epiregulin are associated with a several CID, such as cancer, asthma and other pulmonary diseases, and Crohn’s disease [85,96,97,98,99]. Furthermore, inflammation amplifiers, especially IL-6 and epiregulin, cause inflammation and consequently are associated with CID. IH increases the expression of IL-6 and epiregulin in VSMCs, and therefore may increase the incidence of CID in other tissues, as well as in vessel walls.

Martínez-García et al. attempted to analyze the relationship between OSA and cancer but found that the evidence was limited by a number of factors, including inadequately assessed IH, nonspecific cancer sites, and the inclusion of studies designed to serve other purposes. They concluded that the evidence was not strong enough to infer a relationship between OSA and cancer incidence or progression [100]. In contrast, Gozal et al. reported that the presence of OSA may increase the risk of cancer incidence and worsen cancer prognoses [101]. However, the underlying molecular mechanisms of IH in cancer are not fully understood. IL-6 exhibits immune-suppressive effects on T cell-mediated anti-tumor immunity and is well known as a pivotal player in immunosuppressive states in tumor microenvironments, and in the development and metastasis of various cancers such as prostate and ovarian cancers [96,97,102,103,104]. Epiregulin, meanwhile, appears to contribute to the progression of several different human cancers, including bladder, stomach, colon, breast, and other cancers [85]. The IH-induced increases of IL-6 and epiregulin in VSMCs, therefore, let us speculate that IH also causes an increase of IL-6 and epiregulin, and consequently, chronic inflammation in other smooth muscle tissues, resulting in the progression of several cancers. This is consistent with the previously proposed paradigm that activation of inflammation amplifiers is associated with the development of various tumors [105].

IL-6 has been shown to play a pivotal role in the pathogenesis of lung diseases and to act as a key modulator of overall immune response, as well as non-immune cell responses [99]. The up-regulation of epiregulin has also been shown to increase IL-8 production, which contributes to the inflammation and tissue remodeling associated with asthma, bacterial pneumonia, and chronic obstructive pulmonary disease [85]. Given that IH increases IL-6 and epiregulin expression in VSMCs, the same phenomenon may occur in other smooth muscle cells, and thus could contribute to the pathogenesis or progression of diseases in the lungs and airways.

Crohn’s disease is a CID that in 30% to 50% of patients is complicated by intestinal fibrosis and stricture formation as a result of dysregulated wound healing over time. Inflammation, cellular hyperplasia, and increased extracellular matrix production from smooth muscle cells are important factors in the development of fibrosis in patients with Crohn’s disease [106]. Indeed, intestinal smooth muscle cells contribute to fibrosis via production of large amounts of extracellular matrix proteins, cytokines, and growth factors, including IL-6.

Taken together, these facts suggest the possibility that smooth muscle cells in each tissue play pivotal roles in several diseases, including cancer, asthma and other pulmonary diseases, and Crohn’s disease. Therefore, in patients with OSA, IH could contribute to the progression of these diseases at a molecular level.

## 7. Summary and Perspective

OSA, which is characterized by IH, is a highly prevalent respiratory disorder associated with morbidity and mortality from cardiovascular diseases. Atherosclerosis is a major chronic inflammatory cardiovascular disease that is characterized by an excessive proliferation and migration of VSMCs in lesions and plaques. Therefore, research into the intracellular mechanisms underlying IH in VSMCs can provide new insights that may help to establish effective preventive methods for cardiovascular diseases in patients with OSA.

Over the past decade, in vitro and in vivo models of IH have demonstrated intracellular responses to IH in several tissues and cells. In almost all in vitro studies, the effects of IH are compared with that in normoxia or SH. However, misgivings about the definition of hypoxia have recently arisen, because 21% O_2_ as normoxia in the in vitro model of hypoxia represents hyperoxia. It is thought that few studies of IH, including our previous studies, describe how to define normoxic and hypoxic conditions. For better investigation of IH, it is important to confirm the normoxic and hypoxic conditions in cell culture media based on PO2 or SpO_2_ of blood in healthy human, patients with OSA and in vivo study as well as describing the setting of those conditions.

ROS and the transcriptional factors HIF-1 and NF-κB are commonly thought to regulate the inflammation and adaptation of tissues and cells in IH. However, the precise involvement of ROS and the predominant transcriptional factors in VSMCs due to IH have not yet been uncovered. We previously confirmed that IH induces the activation of NF-κB in RASMCs [34], but our promoter assay revealed that the transcriptional factors, such as NF-κB or HIF-1, may not be involved in the IH-induced increase of epiregulin mRNA. The mechanisms underlying IH-induced epiregulin increases thus require further investigation. However, did demonstrate that IH causes the proliferation of VSMCs, and that this is mediated by epiregulin, which in turn is up-regulated via IL-6 [34,35]. Given this, IL-6 amplifier, IL-6, and epiregulin may be key modulators of inflammation in vessels and other tissues in patients with OSA. Therefore, additional research is needed to establish the mechanisms that underly the responses of VSMCs and other smooth muscle cells to IH, and to yield novel therapeutic and prophylactic targets for CID, including cardiovascular diseases in patients with OSA.

## Abbreviations

Akt	Protein kinase B
CID	Chronic inflammatory diseases
CPAP	Continuous positive airway pressure
DUSP1	Dual specificity protein phosphatase 1
EGF	Epidermal growth factor
erbB	EGF receptor
ERK	Extracellular signal-regulated kinase
GLUT	Glucose transporter
hCASMC	Human coronary artery smooth muscle cell
HIF	Hypoxia-inducible factor
IH	Intermittent hypoxia
I-κB	Inhibitor of NF-κB
IKKβ	I-κB kinase-β
IL	Interleukin
MAPK	Mitogen-activated protein kinase
NF-κB	Nucleus factor-κB
NRF2	Nuclear factor (erythroid-derived 2)-like 2
OSA	Obstructive sleep apnea
PHD	Prolyl hydroxylases
RASMC	Rat aortic smooth muscle cell
ROS	Reactive oxygen species
SOD	Superoxide dismutase
STAT3	Signal transducer and activator of transcription 3
VSMC	Vascular smooth muscle cell
